# Using Attenuated Total Reflection–Fourier Transform Infra-Red (ATR-FTIR) spectroscopy to distinguish between melanoma cells with a different metastatic potential

**DOI:** 10.1038/s41598-017-04678-6

**Published:** 2017-06-29

**Authors:** Refael Minnes, Mati Nissinmann, Yael Maizels, Gabi Gerlitz, Abraham Katzir, Yosef Raichlin

**Affiliations:** 1 Department of Physics, Faculty of Natural Sciences, Ariel University, Ariel, Israel; 2 School of Physics and Astronomy, Sackler Faculty of Exact Sciences, Tel Aviv University, Tel Aviv, Israel; 3 Department of Molecular Biology, Faculty of Natural Sciences, Ariel University, Ariel, Israel

## Abstract

The vast majority of cancer related deaths are caused by metastatic tumors. Therefore, identifying the metastatic potential of cancer cells is of great importance both for prognosis and for determining the correct treatment. Infrared (IR) spectroscopy of biological cells is an evolving research area, whose main aim is to find the spectral differences between diseased and healthy cells. In the present study, we demonstrate that Attenuated Total Reflection Fourier Transform IR (ATR-FTIR) spectroscopy may be used to determine the metastatic potential of cancer cells. Using the ATR-FTIR spectroscopy, we can identify spectral alterations that are a result of hydration or molecular changes. We examined two murine melanoma cells with a common genetic background but a different metastatic level, and similarly, two human melanoma cells. Our findings revealed that higher metastatic potential correlates with membrane hydration level. Measuring the spectral properties of the cells allows us to determine the membrane hydration levels. Thus, ATR-FTIR spectroscopy has the potential to help in cancer metastasis prognosis.

## Introduction

Metastasis causes most of cancer related deaths^1^. When choosing treatment for a cancer patient, it is extremely important to correctly identify the metastatic potential of the tumor. Currently, prognosis of metastasis is based mainly on lymph node status and morphological classification of the tumor. Unfortunately, these predictors are limited or inaccurate in many cases, such as breast cancer and pancreatic cancer^2–4^. An alternative method to diagnose metastases is based on genetic testing of the tumor, a costly and time-consuming technique. Moreover, the genetic approach requires prognostic markers that have not yet been identified in all types of cancer^5^. Hence, the goal of this study is to propose a new diagnostic approach for identifying the metastatic potential of cancer cells based on infrared spectroscopy. This approach will offer immediate and patient-specific results that will help to tailor the precise treatment for each patient.

Absorption spectroscopy of biological tissues and fluids in the middle infrared (mid-IR) range of 3–25 µm, is an extremely useful tool for examining the structure and chemical nature of molecules such as phospholipids, proteins, nucleic acids and carbohydrates and their relationship with surrounding molecules^6, 7^. In particular, mid-IR absorption spectroscopy has been found to be a potent tool in the investigation of biological cells, *in vitro* and *in vivo* ^8, 9^ with the potential to distinguish between different types of cells^10^. In particular, it has been suggested that IR spectroscopy could be used for the early detection of cancer cells^11, 12^.

One of the main difficulties of using mid-IR spectroscopy for biological applications is the presence of water in biological tissues and fluids. Water has a very strong absorption over a broad range in the mid-IR and it masks the absorption of other components of the tissue. Therefore, many of the experiments reported in the literature were carried out on dry biological samples^13, 14^. Unfortunately, the IR absorption spectra of dried cells differ from the IR spectra of cells in their natural aqueous state^9, 15–18^. A way to overcome this problem is to use Fourier Transform IR (FTIR) spectrometers, equipped with an Attenuated Total Reflection (ATR) element. This technique enables the study of mid-IR absorption spectra of live cells in solution (i.e. in their unfixed, hydrated state)^19–22^.

**Figure 1:**
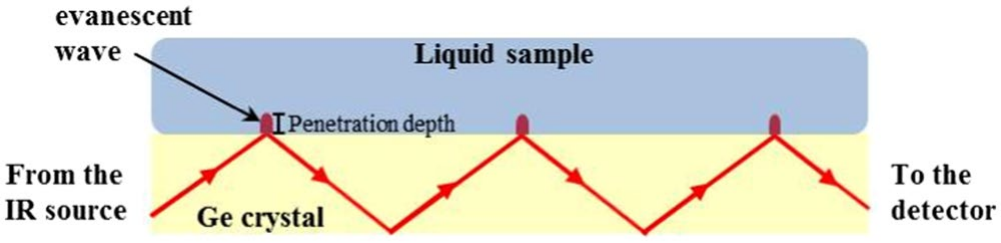
An illustration of the setup of an ATR element with a sample.

The ATR infrared spectroscopy utilizes the TIR (total internal reflection) phenomenon, as illustrated in Fig. 1: An IR beam enters the ATR element (e.g. a Ge ATR crystal) at a certain angle, corresponding to the critical angle between the ATR element and the sample. The beam will undergo TIR and will be reflected several times within the crystal. The internal reflection creates an evanescent wave that extends beyond the ATR element. Since the evanescent wave decays exponentially with distance from the interface, the penetration depth will be a fraction of its wavelength. If a sample is in close contact with the ATR element, the evanescent wave will lose energy at frequencies identical to the sample’s absorbance. The resultant beam can be used to generate the absorption spectrum of the sample. This powerful technique provides a direct way of measuring the mid-IR absorption spectra of samples in contact with an ATR element. Possible samples include: biological cells, biological fluids or sub-cellular components^23^.

Previous studies showed that the higher motility of metastatic cells can be associated with the fluidity of the cell’s membrane^24–28^. This was also found in the well-established mouse model for tumor progression in melanoma, the B16 cell lines^27, 29^.

Here we show that using the ATR-FTIR system to measure the interaction of the cell membrane with water we can distinguish between different malignant stags of human and mouse melanoma cells. These results indicate on the potential of using ATR-FTIR to diagnose the tumor stage of skin cancer cells rapidly.

## Results

### ATR-FTIR spectra of live cells

**Figure 2:**
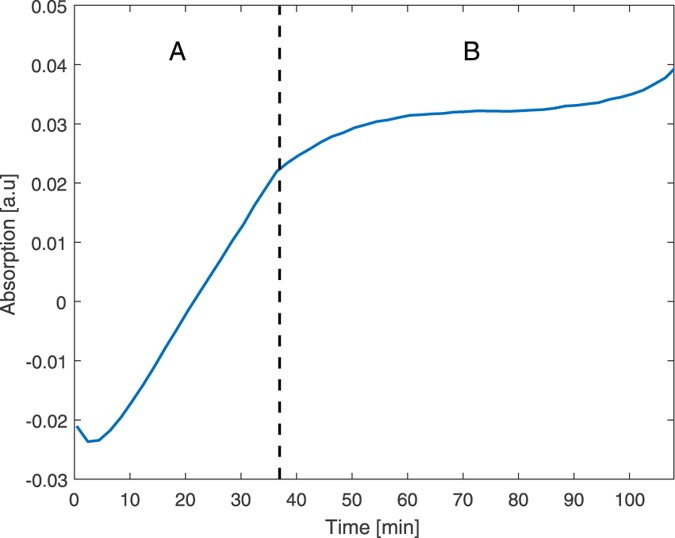
As a demonstration of the different stages during the experiment, the trace shows the absorption of B16-F1 cells versus time at 1540 cm^−1^. The dashed line marks the transition between the sedimentation stage (**A**) and the saturation stage (**B**).

To evaluate the ability of ATR-FTIR to distinguish between similar tumor cells with different metastatic potential, we used well established pairs of mouse and human melanoma cells. The B16-F1 and B16-F10 cells originated from the same parental murine melanoma B16 line but were selected for low- and high-metastatic potential, respectively^30^. The WM-115 and WM-266.4 cells are primary tumor and metastatic human melanoma cells originated from the same patient, respectively^31^. We performed IR spectroscopic measurements on live cell solution using Germanium (Ge) ATR element. The cells were suspended in the medium, forming a homogeneous solution. A small amount of this solution was placed on the ATR crystal and a measurement was carried out immediately. This measurement served as the background measurement for all the subsequent measurements. Meaning, that the spectrum of the homogeneous stage was subtracted from each measured spectrum. During the experiment, the cells’ concentration near the Ge crystal (ATR element) increased. As a result, the IR absorption spectrum increased until the ATR element was fully covered with cells and the absorption spectrum reached a saturation value (Fig. 2). We compared the spectra of the different cell lines in the saturation stage.

### The absorption intensity of proteins

Metastatic cells are known to be more motile in the tissue and have a higher level of fluidity of the plasma membrane (AKA cell membrane) compare to less metastatic cells^24–29^. The higher the fluidity of the plasma membrane the higher its hydration level^32–34^. Thus, the membrane hydration level may be a marker for the metastatic capacity of the tumor cell. One indicator for cell membrane hydration level can be the absorption intensity of proteins. IR spectra of proteins provide information mostly about the secondary structure. Nine characteristic bands are observed in proteins: amide A, amide B and amides I–VII, while the most significant ones are the amide I and II^35^.

**Figure 3:**
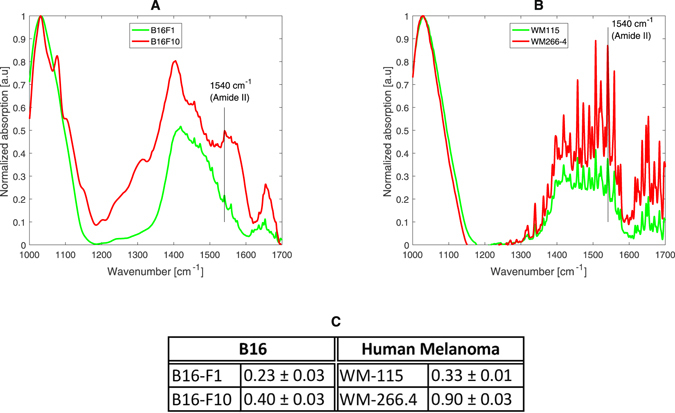
An example of spectra of B16-F1 and B16-F10 (**A**) and WM-115 and WM-266.4 (**B**) cells in the 1000–1700 cm^−1^ range. The graphs present the absorption versus wavenumber at the saturation stage. The normalized intensities of the amide II for the different cell types are shown in panel C.

It was already shown that an increase in the level of hydration will be followed by an increase in the absorption intensity of proteins’ vibration peaks^15^. We assumed that changing the hydration level of the cell membrane will have a similar effect on the membrane’s proteins. A simple way to compare the absorption intensity of a protein-related peak in spectral measurements of different experiments is to normalize its intensity with the intensity of another dominant peak related to a different component. Therefore, for each measurement, we divided the amide II peak intensity (1540 cm^−1^) by the intensity of a strong peak we observed at around 1035 cm^−1^, which can be related to the PO group of phospholipids^7^. We observed that the normalized intensity of the amide II for the murine melanoma cells was 0.23 ± 0.03 for the B16-F1 cells and 0.40 ± 0.03 for the B16-F10 cells. A similar trend was measured for the human melanoma cells with 0.33 ± 0.01 for the WM-115 cells and 0.90 ± 0.03 for the WM-266.4 cells (Fig. 3C). Graphs of the spectral data of the different cell types, showing the differences in the amide II peak intensity, are illustrated in Fig. 3. These results concur with our assumption that the hydration level of the cell membrane can be assessed by changes in the absorption intensity of the membranes’ proteins.

### The intermolecular structure of water molecules

**Figure 4:**
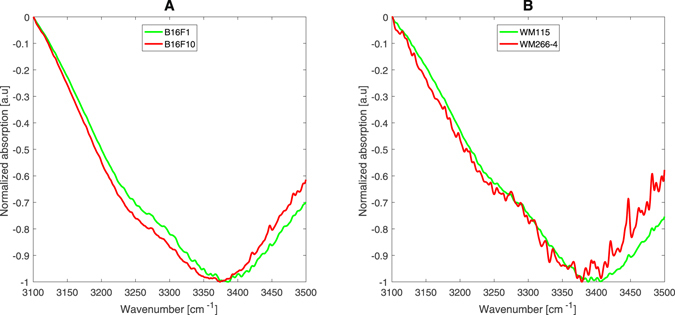
Representing absorption spectra of B16-F1 and B16-F10 cells (**A**) and WM-115 and WM-266.4 cells (**B**), in the 3100–3500 cm^−1^ range.

An additional marker for the cell membrane hydration level is the intermolecular structure of water molecules. Water molecules can be connected by hydrogen bonding to other water molecules forming crystal-like structures (low density water = LDW) or they can be connected to non-water molecules (high density water = HDW)^36–38^. It has been shown that the hydrogen bonding between neighboring water molecules affects the vibration rate of the molecule’s O-H stretch modes and therefore a difference in the absorption frequency of that mode is obtained for the different ‘types’ (or ‘species’) of the water structures. Furthermore, it has been shown that liquid water can be thought-of as being composed of a mixture of these structural species^39^. Each form has its characteristic O-H stretch mode absorption peak; the LDW around 3200 cm^−1^ and the HDW around 3400 cm^−1^. Figure 4 present the differences between the absorption spectra of B16-F1 and B16-F10 cells and WM-115 and WM-266.4 cells, in the 3100–3500 cm^−1^ range.

**Figure 5:**
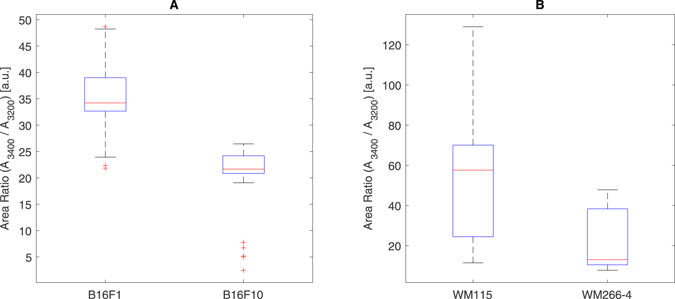
A boxplot illustration of the distribution of the measured values of the ratio between the area of the fitted Gaussian centered around 3400 cm^−1^ and that of the Gaussian centered around 3200 cm^−1^, i.e. the peaks of the HDW and LDW, respectively. The results for B16-F1 and B16-F10 cells are shown in (**A**) and the results for WM-115 and WM-266.4 cells in (**B**). The areal ratio measurements summarized in the plot were done during the saturation stage.

The difference in the hydration level should reflect on the ratio of HDW to LDW (Fig. 5). The reason for it is that as the hydration level is higher (i.e. water molecules are in interaction with a hydrophilic surface), it gets more energetically convenient for the water molecules to break the inter-molecular hydrogen bonds that form the structural order^40^. We based our analysis on a simplified model of the 4-component mixture model presented by Raichlin *et al*.^39^ and used a 2-components mixture. For each measurement, we fit to the absorption spectrum, in the range from 3145 cm^−1^ to 3585 cm^−1^, a mathematical model which consists of a sum of two Gaussians, using a non-linear least square error fit. The model is of the form ${a}_{1}\cdot \exp (-\frac{{(x-{\mu }_{1})}^{2}}{2{\sigma }_{1}^{2}})+{a}_{2}\cdot \exp (-\frac{{(x-{\mu }_{2})}^{2}}{2{\sigma }_{2}^{2}})+C$, where {*a*_1_, *μ*_1_, *σ*_1_, *a*_2_, *μ*_2_, *σ*_2_, *C*} are the fitted parameters. *μ*_1_ and *μ*_2_ were set to be at around 3200 cm^−1^ and 3400 cm^−1^ (actual values were in the ranges 3229–3238 cm^−1^ and 3354–3400 cm^−1^, respectively). Next, we calculated the ratio between the areas of the Gaussian centered around 3400 cm^−1^ and of the Gaussian centered around 3200 cm^−1^, by $\frac{{A}_{3400}}{{A}_{3200}}=\frac{{a}_{3400}\cdot \,{\sigma }_{3400}\,}{{a}_{3200}\cdot \,{\sigma }_{3200}\,}$. Since the background was set at the homogeneous stage, as the cells penetrate the evanescent field, the absorbance of the water is getting smaller and we get a negative peak for the water absorbance. So, the area of each Gaussian is inversely proportional to the number of LDW or HDW molecules in the vicinity of the ATR element. Strictly speaking, if *A*_3400_, *A*_3200_ represent the areas of the Gaussian at 3400 cm^−1^ and of the Gaussian at 3200 cm^−1^, respectively, then $\frac{{A}_{3400}}{{A}_{3200}}\propto {(\frac{{N}_{HDW}}{{N}_{LDW}})}^{-1}$. As we would expect, during the saturation stage we got an approximately constant value for the ratio. Figure 5 presents a boxplot of the areal ratio $(\frac{{A}_{3400}}{{A}_{3200}})$ values measured during the saturation stage, in which the difference between the more metastatic and less metastatic cells is clearly seen. The average areal ratio for B16-F1 cells is 35.2 (s = 5.6) and for the B16-F10 cells is 21.4 (s = 4.7), meaning that B16-F1 cells have more LDW (or less HDW) than B16-F10. Similarly, the average areal ratio for WM-115 cells is 54 (s = 31) and for the WM-266.4 cells is 21 (s = 14), meaning that WM-115 cells have more LDW (or less HDW) than WM-266.4. These results are in accordance with our findings from the absorption intensity of proteins and support our assumption that the hydration level is an important difference between cells with different metastatic levels and that it may function as a tool to determine the metastatic potential of cancer cells.

## Discussion

We described a dynamic ATR-FTIR method for measuring the mid-IR spectra of biological cells. This method allowed us to spectrally analyze the hydration level of the plasma membrane of two variants of the murine B16 cells and two variants of human melanoma and to evaluate their metastatic potential accordingly. We observed two complimentary lines of evidence that relate the metastatic potential of the cells with the hydration level of the plasma membrane. Firstly, there is a higher absorption intensity of amide II for the cells with the higher metastatic potential, which can be explained by the higher hydration level of the plasma membrane. Second, the cells with the higher the metastatic potential had more HDW molecules (or less LDW molecules) found at the cell membrane’s vicinity. These two complimentary tests presented consistent results that may allow us to learn about the metastatic potential of different cells just by analyzing their spectral differences.

In this paper, we used cells in suspension, however, this method can also be implemented on cells taken from tissue (e.g. biopsy), by disrupting the tissue and suspending the cells in solution. Another way that this method can potentially be used is by scanning a suspected tissue with an ATR-FTIR microscope.

The ATR-FTIR method we introduced in this paper has a considerable potential for studying cells in general and especially as a tool for estimating the metastatic potential of cancer cells. The comparison of more metastatic and less metastatic cells by the ATR-FTIR method showed essential spectral differences between the different types of cells, consistent with their metastatic potential differences.

## Methods

### Cell lines

Mouse melanoma B16-F1 and B16-F10 cells were grown in growth medium composed of DMEM (D5796, Sigma-Aldrich, St. Louis MO, USA) supplemented with 10% FCS (04-007-1A, Biological Industries, Beit Haemek, Israel), 0.292mg/mL L-glutamine (03-020-1B, Biological Industries, Beit Haemek, Israel) and 40 units/mL Penicillin-Streptomycin (03-031-1B Biological Industries, Beit Haemek, Israel). Human melanoma WM-115 and WM-266.4 cells were grown in the above medium supplemented with 1 mM sodium pyruvate (S8636 Sigma-Aldrich, St. Louis MO, USA) and non-essential amino acids (01-340-1B, Biological Industries, Beit Haemek, Israel).

B16-F1 and B16-F10 cells were generated from the parental murine melanoma B16 line by isolation of lung metastases cells following their injection into C57 mice. The B16-F1 cells are lung metastases B16 cells. The B16-F10 are lung metastases B16 cells that were repeatedly isolated and re-injected into mice for ten rounds of selection. This selection method generated two cell lines with common genetic background but different metastatic potential: the B16-F10 cells are more aggressive than the B16-F1 cells^30^. WM-115 and WM-266.4 cells are human melanoma cells isolated from the primary tumor and a metastasis of the same patient^31^.

### Cell suspension preparation

The cells were washed 1x with PBS (02-023-1A Biological Industries, Beit Haemek, Israel) and then removed with Trypsin EDTA solution B (03-052- 1B Biological Industries, Beit Haemek, Israel). The cells were spun down and re-suspended in normal growth medium in a final concentration of 1 × 10^6^ cells per ml. The cells in the medium solution were spherical and the radius ratio of B16-F10 cells to B16-F1 cells and of WM-115 cells to WM-266.4 cells was 0.94 ± 0.02 and 0.99 ± 0.02, respectively. (diameter measurements were taken with the aid of a Nikon Eclipse TE2000 microscope coupled to a digital CCD camera).

### FTIR-ATR spectral measurements

FTIR measurements were carried out using a FTIR spectrometer (Thermo Scientific, iS 50) equipped with a Ge ATR device (Thermo Scientific, Smart ARK^TM^). The effective dimensions of the Ge crystal are 47 mm × 5 mm. The refractive index of the Ge is 4 and the angle of incidence in our device was 45 degree, generating 12 reflections. The calculated depth of penetration is 0.664 μm and the calculated effective pathlength is 2.59 μm. The radiation from the IR source of the spectrometer was focused into the ATR crystal and the output radiation (from the other side of the crystal) was focused onto a cooled MCT (mercury cadmium telluride) detector. Measurements were carried out in the spectral range 650–4000 cm^−1^. Each spectrum was an average of 64 scans to increase the signal to noise ratio (SNR).

A medium layer of about 0.5 ml containing about ${10}^{6}$ cells per ml in suspension was placed on the Ge ATR crystal. Due to the small penetration depth (less than 0.7 μm), the evanescence wave will penetrate only a fraction of the first layer of cells in contact with the crystal. We found that a cells’ concentration of about ${10}^{6}$ cells per ml results with a saturation of the absorption signal after less than 2 hours. Using the kinetic measurements option in the Omnic software (Thermo Scientific), we measured the spectra of the cells’ solution every 2 minutes for 3 hours. Due to the cells’ higher density relative to the medium they sank on the ATR crystal. As the cells penetrated the sensor’s evanescent wave zone their spectra versus time were displayed.

### Data Processing

All calculations and data processing procedures were performed using Matlab 2016a (Mathworks inc., USA) software.. Curve fitting of a Gaussians-sum model to the spectra were performed using the non-linear least square curve fitting algorithm implemented in Matlab.
